# Knowledge systems approaches for enhancing project impacts in complex settings: community fire management and peatland restoration in Indonesia

**DOI:** 10.1007/s10113-022-01960-w

**Published:** 2022-07-27

**Authors:** Lisa Robins, Lorrae van Kerkhoff, Yanto Rochmayanto, Niken Sakuntaladewi, Sumali Agrawal

**Affiliations:** 1 Fenner School of Environment and Society, Australian National University, Building 141, Linnaeus Way, Canberra, ACT 2601 Australia; 2 National Research and Innovation Agency (formerly Forest Research and Development Center, Ministry of Environment and Forestry), Bogor, Indonesia; 3 Yayasan Tambuhak Sinta, Palangkaraya, Indonesia

**Keywords:** Peatland fire, Impact pathways, Indonesia, Policy, Stakeholder engagement, Stakeholder mapping

## Abstract

Knowledge systems approaches for enhancing the impact of research are well established and tend to focus on the ways in which researchers can adapt their engagement with stakeholders to achieve a better “fit” between research and action agendas. Yet, these approaches are often based on explicit or implicit assumptions of a skilled and willing research team, and stable and well-defined stakeholders, who have consistent and reasonably well-defined needs. This paper discusses how knowledge systems approaches were developed and deployed in the first phase of the *Gambut Kita* (Our Peatland) project on community fire management and peatland restoration in Indonesia (2017–2021). This was a complex project with a large multi-disciplinary team situated across dynamic institutions in Indonesia and Australia, and addressing a politically controversial topic. To capture the diverse experience of the researchers, and to focus on the needs of stakeholders, we developed a sequence of whole-of-project approaches comprising the following: (i) stakeholder mapping exercises at three nested scales combining stakeholder analysis, knowledge systems mapping and impact pathways analysis; (ii) a project coordinating committee of high-level Indonesian policy-makers and policy-influencers; (iii) a stakeholder engagement forum and (iv) online policy dialogues. We demonstrate its effects through the case of developing an Indonesian Peat Fire Danger Rating System (Peat FDRS), as a core project deliverable. Over 4 years, these structured stakeholder engagement processes gave rise to a Peat FDRS Stakeholder Engagement Network (a multi-institutional working group), which is making significant progress in navigating the complexity inherent in realising an accurate Indonesian Peat FDRS.

## Introduction

International research partnerships, especially those between developed and developing country partners, are increasingly recognising the importance of designing research-for-development that meets the needs of the recipient country decision-makers and their communities. Funders are requiring that research teams understand decision-making contexts more fully and incorporate such understanding into strategies and plans for effective knowledge sharing and implementation. This necessitates collaborations that are inherently transdisciplinary, “where scientists engage with stakeholders to ensure that research adequately reflects the context, needs, and perspectives of multiple groups, especially those who will eventually apply the knowledge” (Rodela and Swartling 2019, p. 83). According to INASP (2021), policy engagement should form “part of a transdisciplinary research approach, fostering connections across academic disciplines and including the active participation of a wide range of stakeholders”.

However, researchers are not always cognisant of the social and political contests that shape the ways in which issues are portrayed and understood (Hanemann and Young 2020; Molle 2009; Stone 2012), and that determine who participates and what forms of knowledge are deemed legitimate (Blomquist 2012, 2020). This is especially difficult in cross-cultural contexts, and where the relevant decision-making bodies are numerous, complex, cross-scale and constantly changing. Applying knowledge systems approaches to understanding the social and political context of decision-making offers a way to systematically explore, understand and engage with these complex decision-making environments. Some well-known and extensively deployed examples of knowledge systems approaches include stakeholder analysis (Reed et al. 2009) and knowledge systems mapping (e.g., Davila et al 2016, Dearden et al. 2003; Manuel-Navarrete and Gallopín 2011; van Kerkhoff and Szlezák, 2006), impact pathways analysis (e.g., Alvarez et al. 2010; Mayne and Stern 2013) and deliberative dialogues (e.g. van de Kerkhof 2006; Dryzek and Niemeyer 2010). New approaches also continue to emerge (e.g., three types of knowledge tool (Swiss Academies of Arts and Sciences no date)).

This paper presents a sequence of approaches that was purposefully designed and deployed to enhance the impact of the first phase (2017–2021) of a bilateral transdisciplinary research-for-development project, *Gambut Kita* (Our Peatland), between the governments of Australia and Indonesia. The project aimed to support the Government of Indonesia in its efforts to protect and restore peatlands through identifying improved community fire management and sustainable livelihood options that reduce the number and intensity of peat fires (Mendham et al. 2016). As a project that aspired to transdisciplinarity, there was a keen willingness to engage with peatland decision-makers, from community (village) scale through the various administrative levels to national government agencies and policy-makers. Taking Pohl et al.’s conceptualisation of transdisciplinary research through 10 steps, the steps 2–4 relate to identifying, understanding and engaging with a “societal problem” (following Step 1 which refers to classifying the research as basic, applied or transdisciplinary) (Pohl et al. 2017).

- Distinguish between research question and societal problem; make links between both
- Specify the societal problem identified in step 2 and relate it to the policy cycle (problem framing/policy development/implementation/evaluation; Fig. 2, p. 46)
- Identify knowledge needed by (primary) target group(s); check whether the knowledge needed is what research may provide
- Identify disciplines and societal actors to be involved in the research project. Clarify the role of societal actors and disciplines vis-à-vis your own research (question); identify paths of interaction (informing, consulting, co-producing)

However, in this complex socio-political setting, it was often unclear whose “societal problem” should be the main focus given that, for example, the “problems” of peatland restoration and fire reduction experienced by communities at the village scale were very different from those at provincial or national levels. Similarly, even within scales, there were often different interests that were not well articulated or understood. As a result of the complex and multi-layered stakeholder context, understanding that complexity and the role and position of the *Gambut Kita* project within it was therefore a prior step that needed to be conducted to enable the project to achieve its engaged, transdisciplinary goals.

In this paper, we first give background context on peatland fires and the *Gambut Kita* project, then outline a suite of knowledge systems approaches applied at the whole-of-project level and discuss impacts in the specific case of developing an Indonesian Peat Fire Danger Rating System (Peat FDRS).

## Why manage fire in Indonesia’s peatlands?

**Fig. 1:**
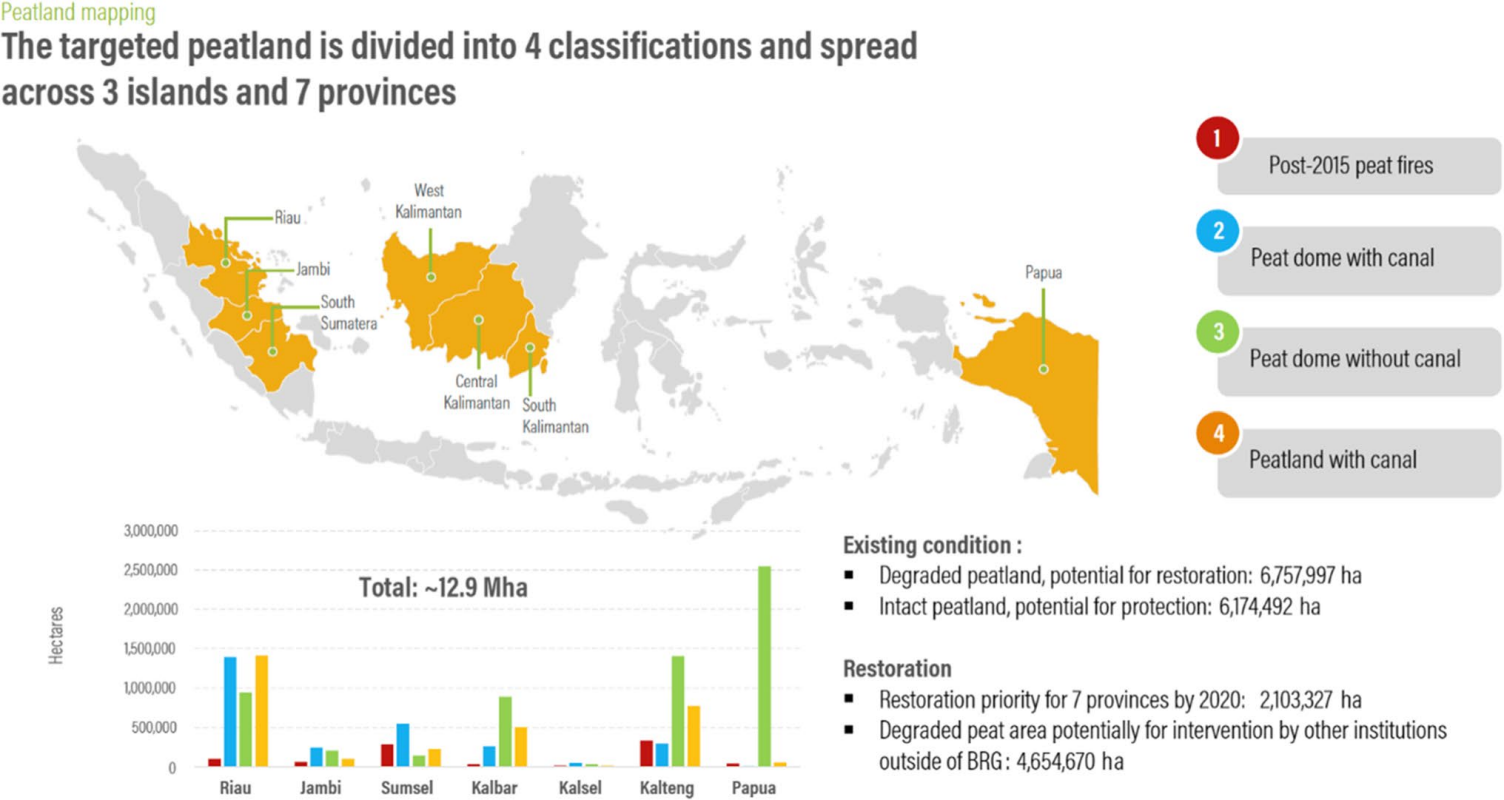
Peatland restoration plan of Indonesia’s Peat Restoration Agency (Wijaya et al. 2017, p. 12)

“Peatlands” are natural areas characterised by peat soils, which comprise partially decayed plant material that has accumulated under waterlogged conditions (Page and Hooijer 2016). Up to 20 times more carbon is stored in tropical peat soils compared to non-peat mineral soil (Harris and Sargent 2016). Peatlands have three components: the peat itself, the water close to the surface which conserves the peat and the unique vegetation that generates the peat soils. Indonesia has approximately 13.43 million hectares of peatlands, found mainly in Sumatra (5.85), Kalimantan (4.54) and Papua (3.01) (Anda et al. 2021). Fire continues to be used to convert and manage Indonesia’s peatland for agriculture and commercial forestry, especially oil palm plantations. Peatland burning is part of a broader pattern of fire use in Indonesia for land clearing and preparation, land acquisition and “as a mechanism to force inhabitants off the land” (World Bank 2016, p. 3). In 2016, President Joko Widodo imposed a permanent moratorium on peatland exploitation and prohibited its burning (Government Regulation No. 57/2016 on Protection and Management of the Peat Ecosystem) and established the Peat Restoration Agency (*Badan Restorasi Gambut*, BRG) (Presidential Decree No. 1/2016). BRG was mandated to restore approximately 2.1 million hectares of peatland by 2020 in seven provinces (Fig. 1), with priority given to the restoration of “peatlands burned in 2015, the high-carbon-stock peat dome areas, and peatlands with canals” (Wijaya et al. 2017, p. 11).

Peatlands are at the heart of four major inter-connected challenges faced by Indonesia — excessive greenhouse gas emissions, toxic transnational smoke haze, loss and degradation of peatland ecosystems and unsustainable livelihoods for peatland-dependent communities.

### Excessive greenhouse gas emissions

Peatlands are a major contributor of global greenhouse gas (GHG) emissions through both oxidation processes and burning (Cooper et al. 2020). Of 69 gigatonnes (Gt) of carbon (C) stored in Southeast Asia’s peatlands (0.25 million km^2^), Indonesia’s share is 57 Gt C (82.6%) (Page and Hooijer 2016). Indonesia is a signatory to the 2015 Paris Agreement (UNFCCC 2015) under the 1994 United Nations Framework Convention on Climate Change. In its Intended Nationally Determined Contribution, Indonesia has made an unconditional commitment to reduce greenhouse gases by 29% by 2030 compared to the business-as-usual scenario (Republic of Indonesia undated). Of this 29% reduction, it is anticipated that 17% (i.e., 59% of the target) will be derived through reducing the incidence and severity of forest fires, and through peatland restoration (Wijaya et al. 2017).

### Toxic transnational smoke haze

Hu et al. (2018) describe smouldering peat fires as “the largest fires on Earth in terms of fuel consumption” (p. 293). In Indonesia, smoke haze arises mostly from surface and peat fires in degraded peatlands. Fires on peatlands release three to six times more particulate matter than fires on other soil types, contributing up to 90% of the haze (World Bank 2015). Tacconi (2016) identifies the main causes of forest and peat fires in Indonesia as land clearing by companies and individual small-scale farmers; other livelihood activities; and unintentional, escaped fires — cautioning “(w)hether companies or smallholders cause the most fires is unlikely to be answered in the immediate future” (p. 642). Despite suppression efforts, large-scale peatland fires typically persist until the next wet season when watertables rise (Huijnen et al. 2016; Page et al. 2002). In addition to dramatic adverse impacts on local communities, transnational haze has created tensions with several South East Asian nations, especially in response to tangible and costly public health and economic consequences (Dohong 2016; Putra et al. 2019; Tacconi 2016). Indonesia alone incurred more than US$16 billion in total economic costs from the 2015 fires; more than twice that of the 2004 tsunami (World Bank 2016).

### Loss and degradation of peatland ecosystems

About 50% of peatlands in Sumatra and Kalimantan have been converted to smallholder agriculture and industrial plantations (of which 64% of the industrial plantation area is oil palm), with only 7% remaining as pristine peat swamp forests (Hergoualc’h et al. 2018 citing Miettinen et al. 2016). Large areas of pristine peatland can still be found in Papua. Peatland systems are “highly resistant to fire in their undisturbed state” (Tacconi 2016, p. 640), as their high moisture content provides natural protection from burning (Moreno et al. 2011; Turetsky et al. 2015). Research on fire-resistant primary tropical forest cover in Sumatra and Kalimantan by Nikonovas et al. (2020) concluded that “fires did not penetrate undisturbed primary forest areas deeper than two kilometres from the forest edge irrespective of drought conditions”, but that “fire-resistant forest now covers only 3% of peatlands and 4.5% of non-peatlands” (p. 1). While fires “within intact peat swamp forests are thought to be rare events” (Cole et al. 2019, p. 1), human activities (logging, land clearing, etc.) and climate change are drying peatlands in many places, increasing their susceptibility to fire (Page and Hooijer 2016; Page et al. 2002; Turetsky et al. 2015). Man-made drainage canals that dissect the peat have resulted in “excessive drainage, subsidence, irreversible drying, loss of habitat and increased risk, frequency and severity of fire… [and] provided easy access for people, especially illegal loggers” (Ritzema et al. 2014, p. 12 citing Diemont et al. 2002; Page et al. 2002). In Kalimantan, Ritzema et al. (2014) report main canals measuring 15–20 m wide and 3–4 m deep, off which a network of secondary and tertiary canals further dissects the landscape. The first step in restoring peatland is rewetting, which principally involves blocking these canals in order to raise the watertable (Ritzema et al. 2014), but this has not yet been attempted in tropical regions on a large scale. Even where efforts to rewet succeed, significant impediments to restoration remain from irreversible peat loss through subsidence (Wösten and Ritzema 2001), and changes to soil chemistry and biology (e.g., Sazawa et al. 2018).

### Unsustainable livelihoods for peatland-dependent communities

Smallholders, communities and industries have long utilised peatlands for agriculture, palm oil production and/or forest plantations, which has contributed significantly to Indonesia’s economy at local to national scales (e.g., Hergoualc’h et al. 2018; Vel et al. 2016). Prior to the permanent moratorium on peatland exploitation and associated prohibition on peatland burning (Government Regulation No. 57/2016), central government policies have supported the clearing and draining of shallow peatland for agricultural and industrial development. Its transmigration programmes have also settled communities, mostly as subsistence farmers, on previously uninhabited peatlands, where indigenous inhabitants occupy neighbouring lands with better-quality mineral soils. For transmigrant families, tenure insecurity and land disputes with Indigenous peoples and business enterprises are commonplace despite resettlement under government schemes (Human Rights Watch 2021). Eilenberg (2021) argues that “environmental regulations, like the central government’s fire ban, have further accelerated the existing smallholder transition from swidden farming and other livelihoods towards large-scale commercial agriculture by putting stress on communities and criminalising traditional practices” (p. 23). However, peatland-based livelihoods are precarious — the peat becomes unsuitable for any form of aerobic or dryland agriculture over a period of 20–30 years (Biancalani and Avagyan 2014; Silvius 2016). There are few options for peatland-dependent communities confronting the challenges of an unsustainable system coupled with the ban on burning (Tacconi 2016). Not burning residue has implications for paddy and other annual crops but is less relevant for perennials (Uda et al. 2020), while viable cropping systems for restored peatlands have yet to be fully developed (Hergoualc’h et al. 2018; Tan et al. 2021; Uda et al. 2020).

## *Gambut Kita* project

A snapshot of the first phase of *Gambut Kita* (2017–2021) is outlined here, together with the case study regions and the core research partners in Indonesia and Australia. This background contextualises the nature and scale of the research-for-development effort, and of the stakeholder engagement challenge.

### Overview

*Gambut Kita* is a bilateral aid project between the governments of Indonesia and Australia. It supports the Government of Indonesia in its efforts to protect and restore peatlands through identifying improved community fire management and sustainable livelihood options that reduce the number and intensity of peat fires. In its first phase (c. AU$4 million), it explored the following five aspects of peatlands:

- *Fire research*: to investigate fire behaviour in peatlands in order to improve existing fire detection and early warning systems for preventing and controlling fires
- *Livelihoods research*: to identify profitable alternative livelihoods on rewetted peatland for smallholders currently deriving their livelihoods on drained peatland
- *Soils research*: to broaden the knowledge base about wet, drained and rewetted peat soils in order to develop better soil and water indicators of peatland condition
- *Policy research*: to inform policy development and implementation at various levels in order to reduce fire occurrence and
- *Knowledge systems research*: to identify and connect key stakeholders to the project’s research components

**Fig. 2:**
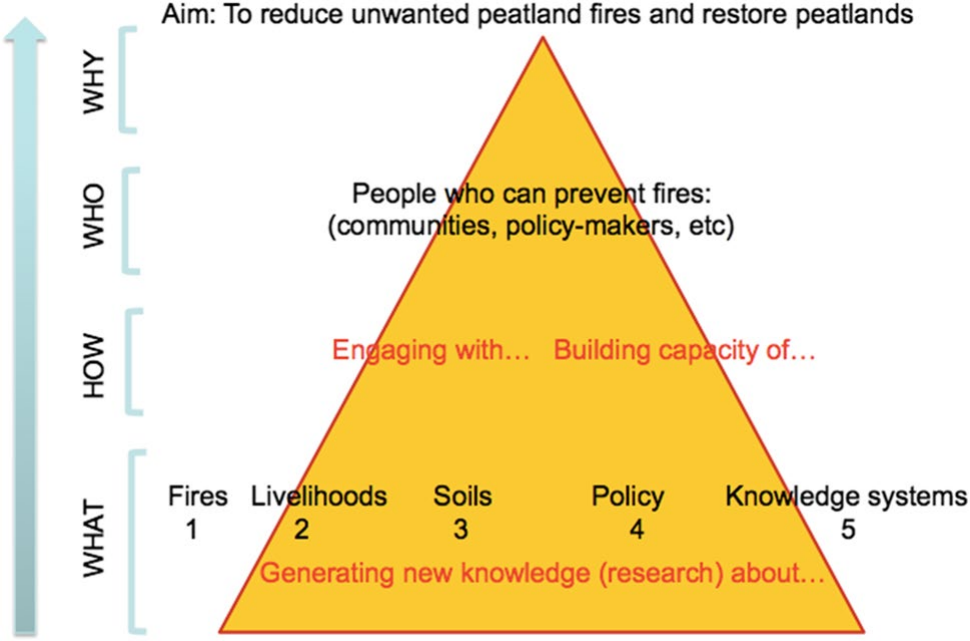
Conceptual diagram showing the relationship between the project’s research and its engagement and capacity building efforts in meeting the project’s overall aim

Figure 2 illustrates the connection between the generation of knowledge by the project team in each of the five arenas and a strategic focus on engaging with and building the capacity of people who can prevent fires in order to achieve the project’s overall aim.

### Case study regions

**Fig. 3:**
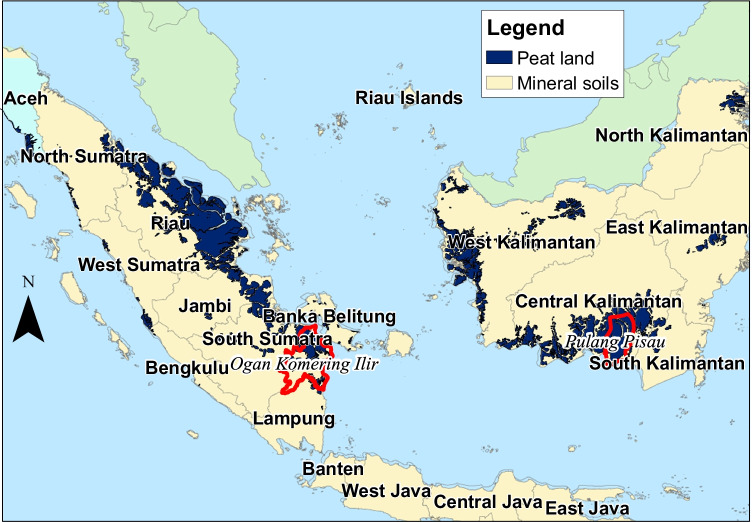
Location of case study regions

*Gambut Kita’s* field work focused primarily on two villages in each of two regions, namely Ogan Komering Ilir (OKI) district in South Sumatra and Pulang Pisau district in Central Kalimantan (Fig. 3), as regions designated by BRG as “highest priority” for peatland restoration (BRG 2016a, b).

South Sumatra (91,590 km^2^) is one of ten provinces in Sumatra (SIMATA 2020), of which approximately 10% is peatland (Miettinen and Liew 2010; Putra et al. 2019). OKI (18,360 km^2^) is one of its 21 administrative districts (17 regencies and 4 municipalities) (SIMATA 2020). Just over half the area of peatland in South Sumatra prioritised for restoration by BRG is in OKI district (640.5 km^2^ of 1206 km^2^) (BRG 2016a). Kayu Labu, a village assessed as very vulnerable to peat fire, was selected as *Gambut Kita’s* primary focal village for South Sumatra.

Central Kalimantan (153,560 km^2^) is one of five provinces in Kalimantan (Indonesian Borneo) (MoEF 2015), of which almost 18% (27,000 km^2^) is peatland. Pulang Pisau is one of its 13 administrative districts. Of the 10 districts prioritised for peatland restoration by BRG, Pulang Pisau, has the largest area at 660,140 ha, of which 164,697 ha is categorised as first priority (BRG 2016b). The project’s primary focal village, Tumbang Nusa, is proximate to the Tumbang Nusa Peat Research Forest Area operated by the Forestry and Environmental Research Development and Innovation Agency (FOERDIA).

### Research partners and their contributions

Indonesia’s lead agency for the first phase of *Gambut Kita* was FOERDIA in the Ministry of Environment and Forestry (MoEF). In May 2021, FOERDIA was consolidated into the National Research and Innovation Agency (*Badan Riset dan Inovasi Nasional*, BRIN) (Presidential Decree No. 33/2021). Field-based activities in the two case study regions were mostly conducted with and through their (former) research offices (*Litbang*) in Palembang (South Sumatra) and in Banjarbaru (located in South Kalimantan, but also administering Central Kalimantan). The current process of institutional restructuring has yet to determine the position of regional *Litbang* offices.

*Gambut Kita’s* three Indonesia-based non-government organisation (NGO) and tertiary sector research partners were based in Palangkaraya, the capital of Central Kalimantan, namely Borneo Orangutan Survival Foundation (BOSF), Yayasan Tambuhak Sinta (YTS) and the University of Palangaka Raya (UPR).

Core Australian partners for the first phase were the Commonwealth Scientific and Industrial Research Organisation (CSIRO), as the project’s overall lead, and four universities: Australian National University (ANU), University of the Sunshine Coast (USC), RMIT University and James Cook University (JCU).

The project team comprised 80 members in total, of which 52 were based in Indonesia (30% female) and 28 in Australia (54% female). The contribution of Indonesia-based partners represented 8.4 full-time equivalent (FTE) positions, of which 7.4 FTE was FOERDIA as the lead in-country agency. Of the Indonesia-based team, 34 were located in the target regions, with 12 in Palembang and 22 in Banjarbaru and Palangkaraya. Australian partner contributions represented 6.4 FTE positions, of which 1.7 FTE was CSIRO as the commissioning institution.

## Knowledge systems approaches for enhanced impact

**Fig. 4:**
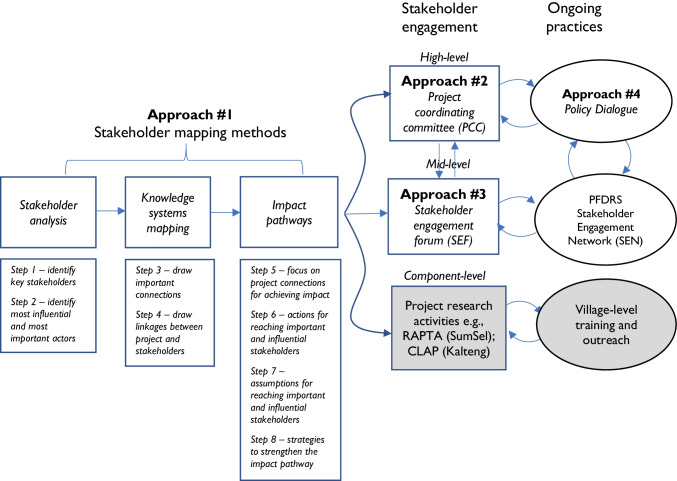
*Gambut Kita’s* core package of knowledge systems approaches applied across all project components (shown in italics). Notes: CLAP, community-led analysis and planning; Kalteng, *Kalimantan Tengah* (Central Kalimantan); KM, knowledge management; PFDRS, Peat Fire Danger Rating System; RAPTA, resilience, adaptation pathways and transformation approach; SumSel, *Sumatera Selatan* (South Sumatra); shaded component-level approaches not discussed in this paper

*Gambut Kita* adopted a core package of knowledge systems approaches across all project components, namely (i) stakeholder mapping exercises at three nested scales combining stakeholder analysis, knowledge systems mapping and impact pathways analysis; (ii) a project coordinating committee (PCC) of high-level Indonesian policy-makers and policy-influencers; (iii) a stakeholder engagement forum and (iv) a series of monthly online policy dialogues with the PCC. These approaches were sequential, in that the first mapping activities informed the steps that followed (Fig. 4), and complemented ongoing finer-grained stakeholder engagement at village level (not the subject of this paper), particularly through the CSIRO-led resilience, adaptation pathways and transformation approach (RAPTA) in South Sumatra (Fleming et al. 2021) and YTS-led community-led analysis and planning (CLAP) in Central Kalimantan (Fleming et al. 2021; YTS 2019).

### Stakeholder mapping methods

The methodology used to systematically identify and examine project strategies for engagement towards achieving the project goals comprised an 8-step process, collectively referred to here as “stakeholder mapping exercises”. This process combined stakeholder analysis (Reed et al 2009, Dearden et al. 2003) and knowledge systems mapping (e.g., Davila 2016; Manuel-Navarrete and Gallopín 2011; Van Kerkhoff and Szlezák, 2006) with impact pathway analysis (e.g., Alvarez et al. 2010; Mayne and Stern 2013). These three methods addressed the questions:

- Who are the main actors in the existing peatlands restoration and fire management system, and how are they connected?
- How do these actors create, seek, share, use and apply knowledge; and how do planned activities in the project link with these connections or create new connections?
- What assumptions are made in believing these activities will contribute to the overall goal of the project?

These stakeholder mapping exercises were conducted at three levels, namely:

- International and national level — In Bogor, West Java (10 & 14 September 2018) with FOERDIA team members and Australian members of the knowledge systems research team
- Provincial and district/regency level — In Palangkaraya, Central Kalimantan (11 & 12 September 2018, Fig. 5a) with team members from FOERDIA, *Litbang* Banjarbaru, BOSF, YTS and UPR, and Australian members of the knowledge systems research team; in Palembang, South Sumatra (15 October 2018) led by FOERDIA Bogor knowledge systems research team members with *Litbang* Palembang team members, with supplementary follow-up interviews (16–18 October) and
- Sub-district and village level — as per provincial and district/regency level

**Fig. 5:**
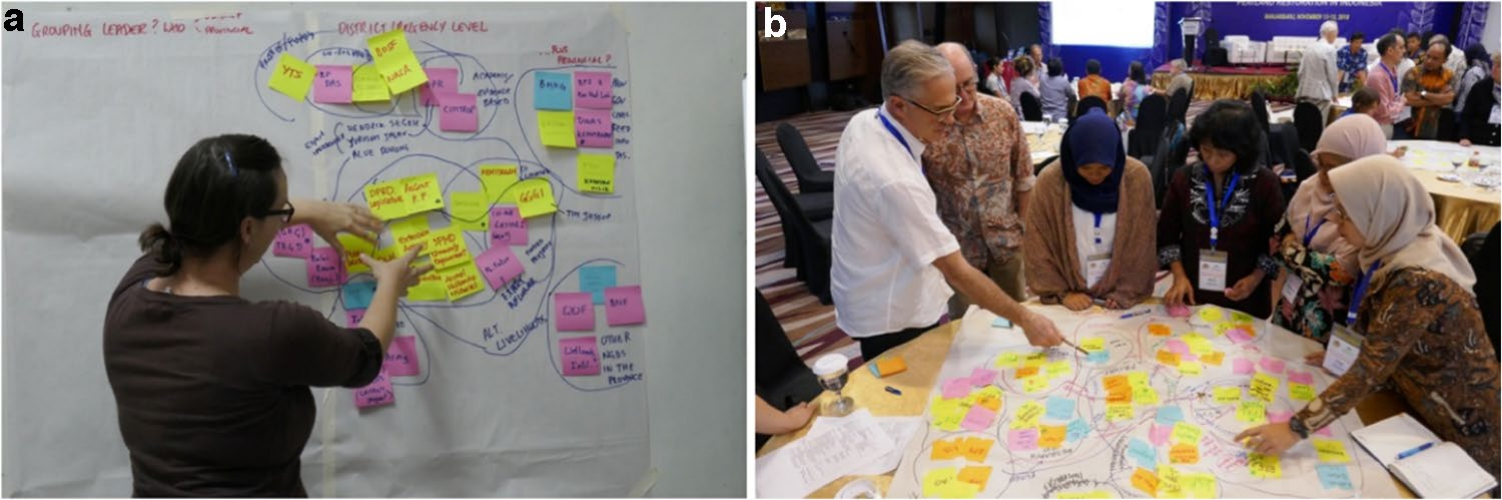
**a** Stakeholder mapping exercise for district/regency level, Palangkaraya, Central Kalimantan, 12 September 2018 (Source: Lisa Robins, ANU). **b** Project team annual meeting, Banjarbaru, Central Kalimantan, 13–15 November 2018 (Source: Lisa Robins, ANU)

The project team deliberated on and refined the resulting stakeholder maps at its subsequent annual project meeting in Banjarbaru, South Kalimantan (13–15 November 2018, Fig. 5b).

The steps are outlined below.

#### *Stakeholder analysis*

Step 1 identified the key stakeholders. Project team small groups brainstormed the relevant stakeholders (organisations, individuals, projects, etc.) at each level, resulting in one map for each of the international and national level; provincial and district/regency level; and sub-district and village level.

Step 2 identified the most influential actors (those who may have enabled or blocked the project achieving its goals) or the most important actors (those who were essential to the project achieving its goals) in each grouping. For example, farmers who set fires may be important in achieving the goals of the project, but may not be very influential in whether the project can progress. These stakeholders were labelled on the map. A limit was set of not more than ten of each for the whole map.

#### *Knowledge systems mapping*

Step 3 involved drawing the important connections between the stakeholders, highlighting where they represented or included knowledge-based processes. Linkages were drawn between the stakeholders on the map with arrows showing connections and the direction of those connections. At this stage, a range of important connections such as funding (funds flow from “A” to “B”) or political influence (organisation “X” can influence organisation “Y”) could be included, but particular attention was given to any knowledge-based connections like technical advice, data provision or sharing, research collaborations and best practice guidelines. For example, FOERDIA staff in *Litbang* Banjarbaru provide technical advice to local and provincial government agencies.

Step 4 placed the project as an entity on the map by drawing any existing or planned linkages between the project and the stakeholders. This was best done at the level of the project’s activities (i.e., specific activities that were intended to include or connect with specific stakeholders) or at the level of its management. An example of the former is village-level surveys that linked the project to the local villages via information sharing. An example of the latter is the team leaders consulting with the Australian Embassy about emerging findings. The activity or output that was intended to connect the project with the stakeholder was noted on the arrows.

#### *Impact pathways*

Step 5 focused on particularly important project activities, and discussing whether, how and to what extent these activities connected the project with important or influential entities on the map to achieve impact. The following impact pathway categories were used to identify:

- Activities — what are you planning to do in your research?
- Outputs — what are the planned outputs from these activities?
- Outcomes — who are the immediate recipients of these outputs? What are you expecting them to do with the outputs you provide?
- Impacts — what are the expected impacts from their actions?

Step 6 identified clear and direct connections between the important and influential organisations. In the absence of clear and direct connections between project activities and important or influential entities, the linkages already noted on the map were considered in terms of pathways that could be followed for the project activities to reach them. Important gaps were identified, including weak links where stronger ones might be needed. For example, social surveys may connect strongly with the important group of villagers who set fires; however, the most influential local actor may be the village customary leader, so will the project activity engage the local customary leader?

Step 6 discussed the strategies, tools or other actions the project team could take to reach the important and influential stakeholders. Consideration was given to whether other teams within the project may be able to facilitate connections, and whether other steps could be taken to reach these actors. For example, the soils team might identify BRG as an important and influential actor, but the policy team may have already established connections with them. Consideration was given to leveraging existing connections to facilitate flows of knowledge and information from the project more generally.

Step 7 asked about the assumptions needed to imagine successfully reaching these important and influential stakeholders. Based on the identified pathways, a “best scenario” of influence was envisaged towards achieving the goals of the project. Assumptions for achieving this impact by influencing these stakeholders were documented, with consideration of:

- How much do you know about whether these are realistic assumptions?
- Are there important assumptions that you do not know enough about? Or that you know are not strong?
- Are there any strategies or steps you can take to find out, or to strengthen weak assumptions?

Step 8 involved the development of strategies to strengthen the impact pathway by addressing weak linkages and uncertain assumptions. Strategies were discussed that could be used to address the weak pathways, or those where assumptions were uncertain. Consideration was given to whether communications targeted these stakeholders or intermediaries along the identified pathway. For example, it is assumed that the customary village head will read and respond to emails; however, as it is not known whether the village head has email, a follow-up call or personal visit could be made.

The workshop activities embodied in Steps 1–8 were designed to collaboratively identify and build our shared understanding of the existing knowledge system. As many of our Indonesian team members were also employees of government or held long-standing relationships with villages, their expertise about the relationships and connections between different organisations and individuals was substantial. By structuring the conversations through these mapping exercises (Steps 1–8), the team was able to identify the next steps of stakeholder engagement that were based on this initial assessment. These included the PCC, the stakeholder engagement forum and the village-level research. Village-level research was suspended over the period reported here due to COVID-19 travel restrictions within Indonesia, and so only the first two will be described.

### Project coordinating committee

On the basis of the stakeholder mapping exercises outlined above, the project leadership team undertook to establish a PCC. This comprised high-level policy-makers and policy-influencers representing Indonesian agencies with key responsibilities for community fire management and peatland restoration, together with a representative of the Australian Government funding agency, Australian Centre for International Agricultural Research (ACIAR), and CSIRO as the Australian commissioning organisation (Table 1). This body formed an important feedback channel, whereby the members of the committee were able to provide input on the direction of the project and its utility for government decision-makers.

**Table 1:** Project coordinating committee members

	Committee member
Indonesia	
Ministry of Environment and Forestry (MoEF)	Head, Center for Socio-economic, Policy and Climate Change Research and Development, Forestry and Environmental Research, Development and Innovation Agency (FOERDIA)^
	Secretary, FOERDIA
	Head, Bureau of Foreign Cooperation
	Director, Land and Forest Fire Control
	Director, Peat Damage Control
	Director, Forest Production Management Unit
	Director, Forest Protection Management Unit
	Director, Social Forestry and Customary Forest Business Development
Peat and Mangrove Restoration Agency *(Badan Restorasi Gambut dan Mangrove*, BRGM; formerly Peat Restoration Agency, *Badan Restorasi Gambut*, BRG)	Deputy, Research and Development (substituted with Head, Research Working Group)
National Development Planning Agency (*Badan Perencanaan Pembangunan Nasional*, BAPPENAS)	Director, Forestry and Water Resources Conservation
Center for International Cooperation in Sustainable Management of Tropical Peatland (CIMTROP)	Director
Local government	Regent, Pulang Pisau District Regent, Ogan Komering Ilir (OKI) District
Australia	
Funding organisation	Research Program Manager, Australian Centre for International Agricultural Research (ACIAR)
Commissioning organisation	Project leader, Commonwealth Scientific and Industrial Research Organisation (CSIRO)

^PCC Chair and lead agency

The PCC was charged with monitoring progress and providing direction in light of any developments that might affect the research. Chaired by FOERDIA, the PCC committed to meeting at least once a year, generally back-to-back with the project’s annual meeting. Meetings were primarily conducted in Indonesian with interpretation services for English speakers. Project team members attended PCC meetings when needed to contribute to specific items, and meeting outcomes were reported back to the project leadership team.

### Stakeholder engagement forum

While the PCC established the high-level connections for influence, the team also designed and implemented a 1-day stakeholder engagement forum (SEF) as an important opportunity to advance the impact pathways for the whole project. The forum was convened in Bogor on 11 September 2019 and, in addition to the project’s core team members, was attended by approximately 30 important and influential mid-level stakeholders from public, private and community sectors, as identified in the earlier stakeholder mapping exercises. The aim of the SEF was to socialise the project with these key actors, learn about their information and knowledge needs and develop a shared understanding of the challenges and opportunities for fire prevention and improved peatland management. The SEF enabled participants to deliberate on and refine the project’s planned outputs, target audiences and intended outcomes, which were subsequently published as a set of fact sheets.

**Fig. 6:**
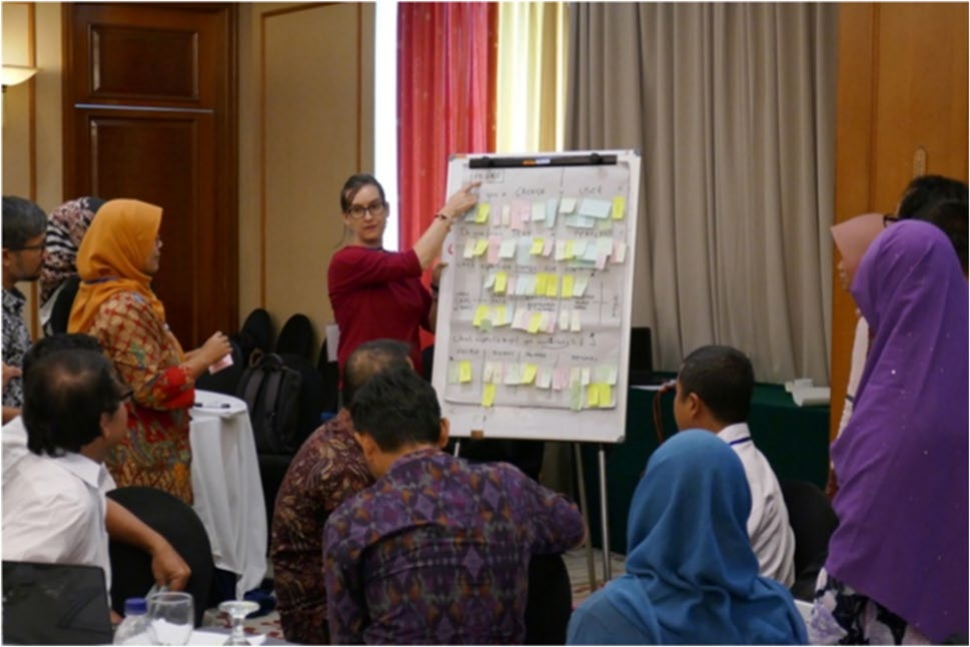
Peat fire danger rating system (Peat FDRS) session, stakeholder engagement forum, Bogor, West Java, 11 September 2019 (Source: Lisa Robins, ANU)

The event was facilitated by a local facilitator with technical expertise in peatland management and conducted in Bahasa Indonesia and English. Team leaders (and/or their most senior researchers) led small-group discussions with their key stakeholders for each of five major project outputs, including an Indonesian Peat FDRS (the case study of this paper, Fig. 6). The intended outcomes were for these influential actors to have greater awareness of the project; and for the project team to develop strategies for targeting outreach and capacity building activities that better met their needs.

### Policy dialogue series

To further consolidate the relationships established with the PCC, a 1-day deliberative dialogue with high-level policy-makers and policy-influencers was planned at the conclusion of the first phase to explore the policy implications of the project’s key research findings. The dialogue was modelled on a successful ACIAR event that took place in Phnom Penh, Cambodia, in 2014, which focused on the implications of rice-based farming systems research for policy in the Mekong region (Robins 2014). The purpose of the dialogue was to encourage reflection on policy coordination, and whether and how the project outputs could be actioned through policy change. Dialogue deliberations were also intended to enable lead researchers to refine the project’s policy-related recommendations. It was anticipated that the dialogue would build legitimacy for realising improved policy settings in the region — through high-level deliberations, through refining and publishing policy briefs, and through networking.

With the onset of the COVID-19 pandemic in early 2020, the project pivoted from a single face-to-face event delivered back-to-back with the project’s 2021 annual meeting, to convening a series of 2–3-h online sessions in dialogue with the PCC and several invited special guests throughout the concluding months of the first phase of the project — “Development of a Peat Fire Danger Rating System for tropical peatlands in Indonesia” (on 23 September) and “Policy options for enhancing the effectiveness of the burning prohibition” (on 9 November), with others to follow in the project’s second phase. A power point presentation on the policy topic was pre-recorded and circulated prior to the dialogue, together with a draft project brief in fact sheet form (Indonesian and English in-design versions). Each dialogue opened with a presentation, after which 2–3 pre-assigned panellists provided their reflections. The dialogue facilitator subsequently opened the event to broader deliberations. Of the 40–50 people online at each dialogue, PCC members or their representatives (refer to Table 1) and the special guests interacted with the relevant project members (authors of the project brief), while a smaller cohort of project team members attended as observers, including those with administrative roles.

## Developing an Indonesian Peat Fire Danger Rating System

While the package of project-wide stakeholder engagement approaches had positive but differential impacts for the project’s five components, here, we examine its impact with respect to *Gambut Kita’s* research on developing a Peat FDRS for Indonesia, as one of the project’s core deliverables. Drawing primarily from *Gambut Kita’s* draft policy brief on developing a Peat FDRS for tropical peatlands in Indonesia (Arifanti et al. 2021), we first outline the imperative for a Peat FDRS and then consider how stakeholder engagement processes have contributed to its development.

### What is a Peat FDRS and why does Indonesia need one?

Fire Danger Rating Systems (FDRS) provide “early warning of the potential for serious fire and haze events… [and] (i)n particular, they identify time periods when fires can readily start and spread to become uncontrolled fires and time periods when smoke from smouldering fires will cause an unacceptably high level of haze” (de Groot et al. 2007, p. 165). Indonesia has an existing FDRS, which was initiated in the late 1990s and based on adapting components of the Canadian FDRS. Multiple Indonesian government agencies have responsibilities for aspects of its management and have contributed variously to its improvement, especially through additional remotely-sensed and ground data.

The existing FDRS, however, is not designed to predict high-risk peat fires. This is highly problematic as the fires on tropical peatland that have caused the most disruption, financial costs and adverse health impacts across the region are those where the peat itself ignited. Going forward, Indonesia’s FDRS needs to be able to distinguish between a non-peatland fire, a peatland fire (surface only) and a peat fire in order to inform targeted and appropriate fire management. An accurate Peat FDRS will require multiple types of remotely-sensed, ground-collected and social data, which will need to be sourced at scale and over time, and require significant capacity and skills across a wide range of disciplines and actors.

### What impact has Gambut Kita had to date?

*Gambut Kita* explored opportunities to further develop Indonesia’s FDRS to better target high-risk peat fires (a Peat FDRS), particularly as a tool for making local communities better aware of the critical times for fire prevention.

The knowledge system approaches described in the “Knowledge systems approaches for enhanced impact” section informed the conduct of the Peat FDRS research. Project team members participated in the stakeholder mapping exercises at the three nested scales. The resulting maps and related information were used to inform the invitation list for the SEF, with particular consideration of influential and important actors. The SEF provided a high-level platform for conducting roundtable workshops with these actors. At the forum, participants agreed to form a Peat FDRS stakeholder engagement network (SEN), notably including agencies with responsibilities for FDRS development in Indonesia, namely Meteorology, Climatology and Geophysical Agency (*Badan Meteorologi, Klimatologi dan Geofisika*, BMKG), BRGM, Directorate General of Climate Change Control (*Direktorat Jenderal Pengendalian Perubahan Iklim*, DJPPI), National Institute of Aeronautics and Space (*Lembaga Penerbangan dan Antariksa Nasional,* LAPAN) and Agency for the Assessment and Application of Technology (*Badan Pengkajian Dan Penerapan Teknologi*, BPPT). Regular 6 monthly events were subsequently held to exchange knowledge and perspectives, identify opportunities and challenges and build the relationships needed to develop and implement a Peat FDRS. In doing so, *Gambut Kita* was able to (i) identify what knowledge and data is needed for a Peat FDRS, (ii) establish that the knowledge and data are mostly available across a diversity of government agencies and (iii) garner their enthusiasm for collaboration.

In the project’s fourth year, preliminary research findings and recommendations were exposed to policy-makers and policy-influencers for feedback through an online policy dialogue on the Peat FDRS with the PCC. A draft policy brief on the Peat FDRS was circulated to the PCC prior to the dialogue (Arifanti et al. 2021), which included specific “policy proposals”. Three agencies — BMKG, DJPPI and Directorate of Peat Degradation Control (*Direktorat Pengendalian Kerusakan Gambut*, KLHK) — were invited to review the draft policy brief and present their perspective at the dialogue. BMKG is the custodian of Indonesia’s existing FDRS (*Sistem Kebakaran Hutan dan Lahan*, SPARTAN). The policy brief was subsequently refined to account for PCC feedback before publishing and promoting via the *Gambut Kita* website (https://gambutkita.org). The Peat FDRS policy brief highlighted two key findings: (i) the importance of a network and better coordination of efforts and resources and (ii) the need to address gaps in the data and relational model. Most significantly, the PCC agreed to support the SEN in guiding the development and implementation of a Peat FDRS for Indonesia. While the development and implementation of a Peat FDRS is a long-term proposition, more formal recognition of the SEN establishes it as a critical platform for Peat FDRS development and subsequent adoption by the Government of Indonesia.

## Discussion and conclusions

While the project started with aspirations to operate in a transdisciplinary way, there were a number of factors that made it very challenging to do so effectively. In the project’s mid-term review (MTR), Sands (2020) described *Gambut Kita* as dealing “with a ‘wicked’ and extremely challenging problem”. For a 4-year research-for-development project of modest budget (c. AU$4 million), we would argue that its agenda was too broad-sweeping and that the following expected outcomes were too far-reaching (ACIAR 2021):

- Increased capacity of the Indonesian Government to restore peatland in a manner that is socially inclusive and biophysically sustainable
- Increased capacity of FOERDIA and other Indonesian partners to research biophysical, economic, policy and social aspects of fire management peatland management and restoration in an inclusive way
- Increased capacity to improve the livelihoods of male and female smallholder farmers on restored peat in the focus areas of the project in South Sumatra and Central Kalimantan
- Reduced peatland burning and fires, leading to reduced smoke haze and greenhouse gas emissions, and a commensurate reduction in negative impacts on public health and local, national, and international economies and
- Improved resilience, communities and industries operating on restored peatland

While the complexity and multi-dimensional nature of the issues were grounds for a transdisciplinary approach, having such a broad mandate was also overwhelming. For each research component (fire, livelihoods, soils, policy and knowledge systems), there exist specialist researchers, a significant body of published and in-progress research, networks of related actors and distinct stakeholder interests. As such, working effectively within any component is a significant undertaking, but endeavouring to also work across them is much harder still. In practice, the project’s modest budget was spread thinly across components, including multiple institutions and field sites located in Pulang Pisau district in Central Kalimantan, and Ogan Komering Ilir (OKI) district in South Sumatra.

Adding further to this complex challenge, *Gambut Kita* started with nine partner organisations, with five based in Australia and four in Indonesia, and added a tenth, University of Melbourne, in 2021. Mostly, these partners had no prior experience of working together. At the project’s outset, there were 80 members in total, of which 34 were located in the focal regions. The project allocation for many individual team members represented a small fraction of their position, which limited their capacity to meaningfully and effectively engage in both component-specific and project-wide activities. In the MTR, Sands (2020) recommended “that research teams should have fewer people with greater time allocations” and argued that the frugal budget is “not helped by the large sometimes loosely managed groups”.

Researchers assigned to a particular component did not always have the disciplinary skills needed to contribute effectively, let alone the capacity to operate across multiple disciplines and incorporating various stakeholder interests and knowledges. This was particularly the case in the two focal regions where FOERDIA staff were assigned to each of the five components, without necessarily having the requisite technical skills (or necessarily the interest), notably for policy and knowledge systems research, but also for project-related engagement and communications. Bammer et al (2020) argue “that some team members must have expertise in research integration and implementation to effectively harness the contributions of the full team” (p. 2).The project did not allocate sufficient resources for those who had that expertise to be able to use it to bridge internal gaps in knowledge or to support greater understanding of transdisciplinary processes. The MTR noted that the knowledge systems team was underfunded in relation to the desired goals of the project and the role the knowledge systems team could play in supporting a more integrated approach, a situation that was not rectified (Sands 2020). While growing capacity in new areas of research was integral to the project, in the context of working remotely with limited resources to travel and spend time together (exacerbated by COVID-19 restrictions), it was difficult to craft or execute work plans that genuinely reflected shared goals.

Working within these broader constraints, we purposefully designed and applied a package of stakeholder engagement approaches across the overall project. While the five components of the project pursued their specific research-for-development activities, these whole-of-project approaches provided common pathways for identifying and engaging with key stakeholders at different scales, informed by a shared understanding of the knowledge system. The first approach — *the stakeholder mapping exercises* — enabled the systematic identification and examination of project’s key stakeholders, their relationships and how the knowledge generated by the project may fit in this complex and dynamic landscape. The second approach — *the PCC* — provided a peak body of high-level policy-makers and policy-influencers for oversighting the project, and entry points for interacting with government agencies strategies for engagement towards achieving the project goals. The third approach — *the SEF* — targeted important and influential mid-level stakeholders arising from the stakeholder mapping exercises in order to socialise the project, learn about their information and knowledge needs and develop a shared understanding of the challenges and opportunities. The fourth approach — *the Policy Dialogues* — enabled high-level deliberations amongst PCC members and lead researchers for realising improved policy settings in the region.

The case example of the Peat FDRS, as one of the project’s core deliverables, demonstrates the value-added of these structured stakeholder engagement approaches, which gave rise to the Peat FDRS SEN and its subsequent recognition by the PCC as a longer-term platform for advancing an Indonesian Peat FDRS. Navigating the complexity inherent in realising a Peat FDRS for Indonesia will depend greatly on this sort of strengthening of cross-agency coordination and collaboration. Enabling the development of an accurate Peat FDRS will enhance Indonesia’s ability to target high-risk peat fires, facilitated by shared understanding of the knowledge system and the project’s role within that dynamic space.
